# From Gut to Eye: Exploring the Role of Microbiome Imbalance in Ocular Diseases

**DOI:** 10.3390/jcm13185611

**Published:** 2024-09-21

**Authors:** Andreea-Talida Tîrziu, Monica Susan, Razvan Susan, Tanasescu Sonia, Octavia Oana Harich, Adelina Tudora, Norberth-Istvan Varga, Dragomir Tiberiu-Liviu, Cecilia Roberta Avram, Casiana Boru, Mihnea Munteanu, Florin George Horhat

**Affiliations:** 1 Department of General Medicine, Doctoral School, “Victor Babes” University of Medicine and Pharmacy, 300041 Timisoara, Romania; andreea.tirziu@umft.ro (A.-T.T.); norberth.varga@umft.ro (N.-I.V.); 2 Department of Ophthalmology, “Victor Babes” University of Medicine and Pharmacy, 300041 Timisoara, Romania; mihnea.munteanu@umft.ro; 3 Centre for Preventive Medicine, Department of Internal Medicine, “Victor Babes” University of Medicine and Pharmacy, Eftimie Murgu Square, No. 2, 300041 Timisoara, Romania; 4 Centre for Preventive Medicine, Department of Family Medicine, “Victor Babes” University of Medicine and Pharmacy, Eftimie Murgu Square, No. 2, 300041 Timisoara, Romania; razvansusan@umft.ro; 5 Department of Pediatrics, “Victor Babes” University of Medicine and Pharmacy, Eftimie Murgu Sq. No. 2, 300041 Timisoara, Romania; tanasescu.sonia@umft.ro; 6 Department of Functional Sciences, “Victor Babes” University of Medicine and Pharmacy Timisoara, Eftimie Murgu Sq. No. 2, 300041 Timisoara, Romania; harich.octavia@umft.ro; 7 Multidisciplinary Doctoral School, Vasile Goldis Western University of Arad, Strada Liviu Rebreanu 86, 310419 Arad, Romania; tudora.adelina-georgiana@student.uvvg.ro; 8 Medical Semiology II Discipline, Internal Medicine Department, “Victor Babes” University of Medicine and Pharmacy, Eftimie Murgu Square 2, 300041 Timisoara, Romania; dragomir.tiberiu@umft.ro; 9 Department of Residential Training and Post-University Courses, “Vasile Goldis” Western University, 310414 Arad, Romania; avram.cecilia@uvvg.ro; 10 Department of Medicine, “Vasile Goldis” University of Medicine and Pharmacy, 310414 Arad, Romania; boru.casiana@uvvg.ro; 11 Multidisciplinary Research Center on Antimicrobial Resistance (MULTI-REZ), Microbiology Department, “Victor Babes” University of Medicine and Pharmacy, 300041 Timisoara, Romania; horhat.florin@umft.ro

**Keywords:** gut, microbiota, dysbiosis, ocular surface microbiota, gut–eye axis, uveitis, glaucoma, age-related macular degeneration, AMD

## Abstract

**Background:** The gut microbiome plays a crucial role in human health, and recent research has highlighted its potential impact on ocular health through the gut–eye axis. Dysbiosis, or an imbalance in the gut microbiota, has been implicated in various ocular diseases. **Methods:** A comprehensive literature search was conducted using relevant keywords in major electronic databases, prioritizing recent peer-reviewed articles published in English. **Results:** The gut microbiota influences ocular health through immune modulation, maintenance of the blood–retinal barrier, and production of beneficial metabolites. Dysbiosis can disrupt these mechanisms, contributing to ocular inflammation, tissue damage, and disease progression in conditions such as uveitis, age-related macular degeneration, diabetic retinopathy, dry eye disease, and glaucoma. Therapeutic modulation of the gut microbiome through probiotics, prebiotics, synbiotics, and fecal microbiota transplantation shows promise in preclinical and preliminary human studies. **Conclusions:** The gut–eye axis represents a dynamic and complex interplay between the gut microbiome and ocular health. Targeting the gut microbiome through innovative therapeutic strategies holds potential for improving the prevention and management of various ocular diseases.

## 1. Introduction

The human gut microbiome, a complex and dynamic ecosystem comprising trillions of microorganisms, has been proven to be a key player in maintaining overall health. Recent advances in metagenomic studies have revealed the gut microbiome’s profound influence on diverse physiological systems, extending beyond the gastrointestinal tract [1]. An intriguing and rapidly evolving area of research focuses on the potential connection between gut microbiome dysbiosis—an imbalance in the microbial community—and the development or progression of various ocular diseases [2,3,4]. 

Once thought to be sterile, the eye is now understood to possess its own distinct microbiome, with the ocular surface microbiota (OSM) acting as a key defense mechanism against infections and diseases [5]. Composed largely of bacteria from the *Firmicutes, Actinobacteria*, and *Proteobacteria* phyla, the OSM plays a role in local immune system development, host metabolism regulation, and pathogen defense [5]. An imbalance in the ocular microbiota can lead to pathogenic overgrowth and inflammation, both locally and systemically [4,5,6].

Recent research has also highlighted the influence of the gut microbiota (GM), primarily composed of the *Firmicutes*, *Bacteroidetes*, *Proteobacteria*, *Actinobacteria*, and *Verrucomicrobia* (*Firmicutes* and *Bacteroidetes* together representing up to 90% of the gut microbiota [7]) phyla, on the development and progression of eye diseases [8]. This has led to the concept of a gut–eye axis, where microbes that are present in the gut can modulate ocular immunity [9]. Consequently, alterations in the gut microbial composition have been linked to various ocular conditions, including uveitis, age-related macular degeneration, diabetic retinopathy, dry eye syndrome, and glaucoma [8,9,10].

This review investigates the potential connection between the gut microbiome and ocular health and the possible role of gut dysbiosis in ocular disease development and progression. Additionally, we will investigate potential therapeutic interventions, such as probiotics, prebiotics, synbiotics, and fecal microbiota transplantation, ultimately contributing to the prevention and management of ocular diseases. 

## 2. Methods

In this review, we conducted a comprehensive search of the literature using relevant keywords alone or in combination (e.g., “gut microbiota”, “dysbiosis”, “ocular diseases”, “uveitis”, “age-related macular degeneration”, “dry eye”, “glaucoma”, “diabetic retinopathy”, “ocular surface microbiota”, “gut–eye axis”, “probiotics”, “prebiotics”, “synbiotics”, and “faecal microbiota transplantation“) in major electronic databases (PubMed, Google Scholar, and Web of Science). We focused on peer-reviewed articles published in English, prioritizing recent studies to ensure our review was as up-to-date as possible. No fixed time period was imposed. The resulting literature was carefully analyzed and incorporated into our narrative review. We also examined reference lists of relevant articles to identify additional studies.

Specifically, we focused our search on the impact of gut microbiota on the following ocular conditions: uveitis, age-related macular degeneration, dry eye disease, glaucoma, and diabetic retinopathy. We included original articles and experimental studies, both in mice and humans, that investigated the relationship between gut dysbiosis and these ocular problems. In addition, we collected information from numerous review articles to provide a comprehensive overview of the current understanding of the gut–eye axis and its implications for ocular health.

Studies were selected based on their relevance to the topic of gut microbiome dysbiosis and ocular diseases, with a particular emphasis on the mechanisms and potential therapeutic implications. The resulting literature was carefully analyzed and incorporated into our narrative review.

## 3. Results

Our comprehensive literature search across multiple databases yielded an initial pool of 26,763 results. However, the majority of these hits stemmed from single keyword searches (e.g., “uveitis”). In contrast, combining keywords (e.g., “gut dysbiosis uveitis”) drastically narrowed the results. Following the removal of duplicates and a preliminary screening based on title examination, 25,973 articles were excluded due to their lack of specificity in addressing the research objectives of this review. This initial screening left a pool of 790 articles for further evaluation.

**Figure 1:**
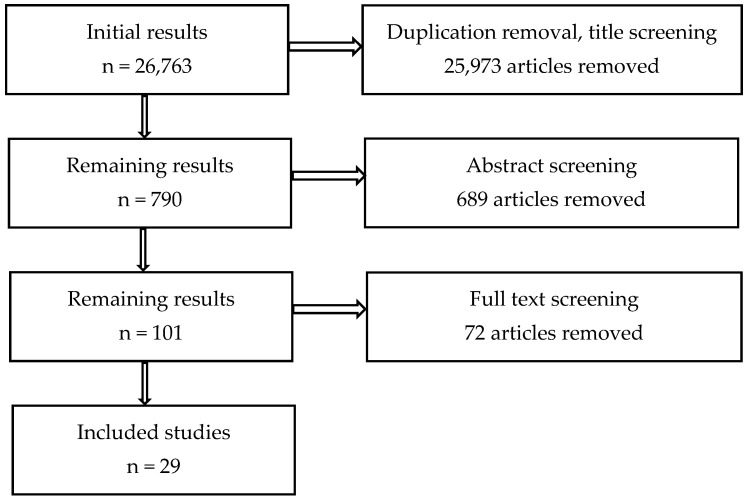
Study selection process.

Subsequently, we conducted a more in-depth assessment of the remaining articles by scrutinizing their abstracts. This process led to the exclusion of an additional 689 articles that did not meet our inclusion criteria, primarily due to their non-experimental, observational cohort, or case-control nature. The final pool of 101 articles was then subjected to a full-text review, during which we further refined our selection to include only original experimental and case-control studies that directly investigated the relationship between gut dysbiosis and the five targeted ocular conditions. This rigorous selection process culminated in a final collection of 29 studies that served as a basis for our review. Figure 1 summarizes our study selection process.

## 4. Gut Microbiota, Functions, and Dysbiosis

### 4.1. A Healthy Gut Microbiota

The gut bacterial microbiome plays a crucial role in human health by aiding digestion, providing protection against pathogens, contributing to immune system development, and producing essential vitamins and short-chain fatty acids (SCFAs) like acetate, propionate, and butyrate. Additionally, it helps maintain the balance of T-cell populations, which is vital for effective immune responses. Inter-individual variations in the gut microbiota may contribute to physiological differences between individuals [1,2,4,8].

**Figure 2:**
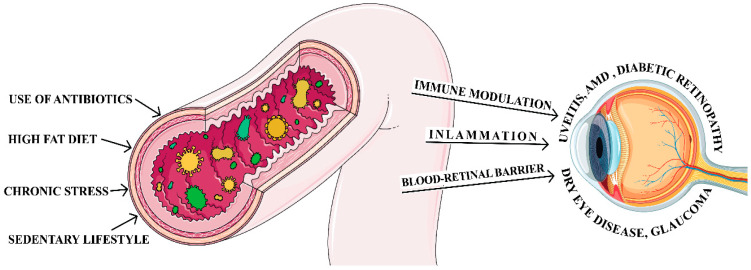
Gut dysbiosis and related ocular diseases. Figure created with Adobe Illustrator 2024 and iStock.

A healthy gut microbiota is typically dominated by a diverse community of bacteria, with *Firmicutes* and *Bacteroidetes* forming the foundation, alongside *Actinobacteria*, *Proteobacteria*, *Fusobacteria*, and *Verrucomicrobia*. The *Firmicutes* phylum encompasses a wide range of bacteria, including beneficial *Lactobacillus* and fiber-digesting *Ruminicoccus*, as well as potentially harmful *Clostridium* and *Enterococcus* species. *Bacteroidetes*, another major phylum, includes *Bacteroides* and *Prevotella*, both key players in carbohydrate breakdown. Other significant phyla are *Actinobacteria*, represented by the probiotic *Bifidobacterium*; *Proteobacteria*, which includes common gut bacteria like *E. coli*; *Fusobacteria*, which includes the *Fusobacterium* species and is often associated with the oral cavity but also found in the gut; and *Verrucomicrobia*, featuring *Akkermansia muciniphila*, which is a mucin-degrading bacteria that resides in the gut mucous layer [1,7,11]. A well-functioning gut microbiota is characterized by a harmonious equilibrium between beneficial microbes, which promote health and fight inflammation and potentially harmful ones that can trigger inflammation and disease. This delicate balance can be upset, however, by various lifestyle and environmental factors, including diets high in unhealthy fats or sugar, lack of physical activity, excessive antibiotic use, and certain underlying health conditions (Figure 2). This disruption, known as dysbiosis, results in changes in the types, numbers, and activities of gut microbes, deviating from a healthy state.

### 4.2. What Causes Dysbiosis?

High-fat diets tend to be rich in saturated and unhealthy fats, which can stimulate the growth of bile-tolerant bacteria like *Bilophila wadsworthia*. These bacteria thrive in the presence of bile acids, which are produced in greater quantities when digesting high-fat foods. *Bilophila wadsworthia* also produces hydrogen sulfide, a compound linked to inflammation and gut barrier disruption [12]. Additionally, high-fat diets may decrease the abundance of beneficial bacteria like *Bifidobacterium*, further compromising gut health. Excess sugar provides a readily available energy source for opportunistic pathogens like *Candida albicans*, leading to their overgrowth. This can create an imbalance in the microbial community and disrupt gut homeostasis. Moreover, excessive sugar intake may reduce the production of short-chain fatty acids (SCFAs) by beneficial bacteria, negatively impacting gut barrier function and immune regulation [13].

Chronic stress triggers the release of stress hormones like cortisol, which can slow down gut motility, reduce the production of the mucus that protects the gut lining, and impair immune function within the gut. This creates an environment less hospitable to beneficial bacteria, allowing opportunistic pathogens to gain a foothold and disrupt the microbial balance. Similarly, a sedentary lifestyle leads to decreased gut motility, resulting in slower transit times for food and waste. This prolonged exposure to the gut environment provides more opportunities for potentially harmful bacteria to colonize and outcompete beneficial microbes. Both stress and inactivity create conditions that favor the growth of detrimental bacteria and compromise the overall health of the gut microbiome, thus contributing to dysbiosis [14].

Antibiotics, while essential for fighting infections, can indiscriminately target both harmful and beneficial bacteria in the gut. This disruption can significantly reduce microbial diversity, leaving empty niches that opportunistic pathogens may readily occupy. Moreover, certain bacteria possess antibiotic resistance genes, allowing them to survive and proliferate in the presence of antibiotics, further skewing the microbial balance. This altered composition can persist long after antibiotic treatment ends, contributing to long-term gut dysbiosis and potentially increasing the risk of various health issues [15].

### 4.3. What Can Dysbiosis Lead To?

The gut microbiota significantly impacts host metabolism by facilitating the breakdown and absorption of complex carbohydrates and fibers that the human body cannot digest alone. It also synthesizes essential vitamins, like B vitamins and vitamin K, which are vital for various metabolic processes. Furthermore, gut bacteria actively participate in bile acid metabolism, influencing lipid and cholesterol levels and potentially reducing the risk of cardiovascular diseases [16].

In addition to its metabolic functions, the gut microbiota plays a pivotal role in immune system regulation. It acts as a training ground for the immune system, teaching it to differentiate between harmful pathogens and beneficial microbes, thus fostering immune tolerance and preventing excessive inflammation. Disruptions to this delicate balance can lead to immune dysregulation, increasing the susceptibility to autoimmune and inflammatory diseases [17].

The gut microbiota also plays a vital role in preserving the effectiveness of the gut barrier, ensuring that harmful substances and bacteria cannot easily pass from the intestines into the bloodstream. Commensal bacteria actively support this barrier by stimulating mucus production and strengthening the tight junctions between intestinal epithelial cells. Dysbiosis, however, compromises this barrier, leading to increased intestinal permeability or “leaky gut”, allowing the translocation of bacterial components and toxins and triggering systemic inflammation [18].

Gut dysbiosis has been linked to various health issues, both within the digestive system (like obesity [19], Crohn’s disease [20], diabetes [21,22], colorectal cancer [23], and gastric cancer [24]) and beyond (such cardiovascular [25] and neurological disorders [26,27] and even depressive-like behaviors [28]). Research suggests this connection might stem from the specific functions of the imbalanced bacteria. However, interpreting these changes in relation to disease is complex and often relies on inferred bacterial functions, sometimes even extrapolating from genus to species level, which requires careful consideration. Our study narrows its focus specifically on the relationship between gut dysbiosis and ocular health, leaving the broader systemic implications beyond the scope of this review.

The ocular diseases potentially influenced by gut dysbiosis include uveitis, age-related macular degeneration (AMD), diabetic retinopathy, dry eye disease, and glaucoma. In the following sections, we will delve deeper into the mechanisms by which gut dysbiosis can contribute to each of these ocular diseases.

## 5. Ocular Surface Microbiota—Composition and Role

The ocular surface is recognized to harbor a diverse community of microorganisms known as the ocular surface microbiota (OSM). This complex ecosystem plays a crucial role in maintaining ocular health by interacting with the host’s immune system and providing a defense against pathogenic invasion.

### 5.1. OSM Composition

The most dominant phyla of the OSM are *Actinobacteria*, *Proteobacteria*, and *Firmicutes*. Within these phyla, common bacterial genera include *Staphylococcus*, *Corynebacterium*, *Propionibacterium*, *Pseudomonas*, and *Streptococcus*. The OSM also harbors a less diverse fungal community, mainly represented by *Ascomycota* and *Basidiomycota*, with *Malassezia* being the most prevalent genus [29]. The epithelial cells of the cornea and conjunctiva typically tolerate the presence of these commensal bacteria without initiating an inflammatory response.

Despite direct exposure to the environment, the ocular surface microbiota (OSM) is distinct from facial and oral microbiota [30]. The existence of a consistent “core” OSM is still debated. Early research relied on culturing techniques, revealing predominantly Gram-positive bacteria like *Staphylococcus*, *Corynebacterium*, *Streptococcus*, and *Propionibacterium*, with less frequent Gram-negative bacteria and fungi [31]. However, culture methods have limitations in detecting the full spectrum of the OSM, prompting the use of 16S rRNA sequencing for more accurate analysis. This molecular approach has expanded our understanding of the OSM’s diversity. Graham et al. (2007) identified a broader range of bacteria using 16S rRNA sequencing compared to culture methods [32]. Subsequent studies proposed different potential core OSM compositions, with varying bacterial genera and phyla identified across different populations. Despite the ongoing debate on a definitive core OSM, there is a clear consensus across studies regarding the most abundant phyla.

The composition of the OSM appears to be intricately linked to the gut microbiota through various potential mechanisms. While no conclusive studies have demonstrated a direct, causal link between gut dysbiosis and specific changes in OSM composition, a growing body of literature suggests indirect pathways and mechanisms through which gut microbiota impacts the composition of the OSM. Labetoulle et al. (2024) describe the gut–eye axis, underscoring the potential impact of gut dysbiosis on the development and severity of ocular surface disorders, particularly dry eye disease (DED). They propose that alterations in the composition of the GM, characterized by a decrease in beneficial bacteria such as *Faecalibacterium prausnitzii* and an increase in pro-inflammatory bacteria like *Escherichia coli*, can trigger a cascade of systemic effects. These include the disruption of intestinal barrier integrity, leading to increased intestinal permeability and the translocation of bacterial products like lipopolysaccharides (LPS) into the systemic circulation. This translocation can activate toll-like receptors (TLRs) on immune cells, promoting the secretion of pro-inflammatory cytokines such as IL-1β, TNF-α, and IFN-γ. Concurrently, gut dysbiosis may lead to a reduction in the production of short-chain fatty acids (SCFAs), which are metabolites known for their anti-inflammatory properties. The resulting systemic inflammation and immune dysregulation can then impact the ocular surface microenvironment, potentially altering the composition and function of the OSM, contributing to the development or exacerbation of DED [33].

A study from Xue et al. (2021) further describes this intricate relationship by exploring the connection between the GM and various ocular diseases, including autoimmune uveitis, age-related macular degeneration, and glaucoma. They emphasize the role of the GM in modulating the host’s immune response, particularly the balance between T-helper cell subsets (Th1, Th2, Th17) and regulatory T cells (Tregs). Gut dysbiosis can disrupt this delicate balance, leading to a pro-inflammatory state that may indirectly influence the composition and stability of the OSM. For instance, a decrease in SCFA-producing bacteria in the gut can lead to reduced Treg cell differentiation and function, potentially promoting a Th17-driven inflammatory response that could impact the ocular surface. These studies collectively underscore the profound influence of the GM on ocular surface homeostasis and highlight the potential therapeutic implications of targeting the GM in the management of ocular surface diseases [34].

### 5.2. The Role of the OSM

These commensal microorganisms engage in a complex interplay with the host’s immune system, contributing to both protection against pathogens and the overall health of the ocular surface.

The OSM contributes to the integrity of the ocular surface barrier. Certain bacteria within the OSM produce bacteriocins, which are antimicrobial substances that can inhibit the growth of other microbes. Additionally, the OSM is involved in regulating mucin production, a key component of the tear film that lubricates and protects the eye. By modulating mucin composition and secretion, the OSM helps to maintain a healthy tear film and prevent dryness and inflammation [31].

The OSM interacts with the ocular surface epithelium and resident immune cells, shaping the local immune response. In a healthy state, the OSM helps to maintain immune tolerance, preventing unnecessary inflammation and promoting a peaceful coexistence with the host. This tolerance is mediated in part through interactions with pattern recognition receptors (PRRs), such as toll-like receptors (TLRs), which can distinguish between harmless commensals and potential pathogens. The ocular surface microbiota can further contribute to a balanced immune response by affecting the levels of T-cells that promote or suppress inflammation [35].

The presence of a diverse and balanced OSM provides a natural defense against pathogenic microorganisms. Through competition for nutrients and space, the OSM can limit the colonization of more virulent microbes. Furthermore, the OSM stimulates the production of antimicrobial substances, such as secretory IgA (SIgA) in tears, which can directly neutralize pathogens. By bolstering the local immune response, the OSM plays a crucial role in protecting the eye from infection [36].

## 6. How Gut Dysbiosis Impacts the Eye

Recent research has revealed a fascinating connection between the gut and the eye, which is referred to as the gut–eye axis. This communication pathway highlights the intricate relationship between the gut microbiome and ocular health [4,5,9,10].

Gut homeostasis contributes to ocular health by regulating the immune system and producing anti-inflammatory factors like short-chain fatty acids (SCFAs) [35]. Disruptions to this balance can promote systemic inflammation, gut barrier dysfunction, and potentially impact the ocular surface and retina [34]. While the precise mechanisms remain under investigation, evidence suggests the gut microbiota (GM) can influence ocular health through immune modulation, maintenance of the blood–retinal barrier, and the production of beneficial metabolites.

### 6.1. Gut Dysbiosis and Uveitis

Uveitis, a sight-threatening inflammatory condition affecting the uvea (the middle layer of the eye), has a complex and multifactorial pathogenesis often rooted in immune dysregulation [36]. Recent evidence suggests a strong association between uveitis and changes in the composition of gut microbiota [37,38].

Studies in both animal models and human patients have revealed distinct patterns of gut microbial imbalance in uveitis. In individuals with the condition, there is a marked reduction in beneficial bacteria known for their anti-inflammatory and probiotic properties, such as *Lachnospira*, *Faecalibacterium prausnitzii*, *Ruminococcus*, *Bacteroides*, and *Bifidobacterium adolescentis* [39]. This depletion may contribute to a heightened inflammatory response, exacerbating uveitis symptoms.

Animal models have provided valuable insights into the causal relationship between gut dysbiosis and uveitis. In experimental autoimmune uveitis (EAU) mice, alterations in gut microbiota composition were associated with increased disease severity [40,41]. Early research in rats genetically prone to uveitis showed altered gut microbiota composition, with a decrease in beneficial bacteria like *Bacteroides vulgatus*. In mice with experimental autoimmune uveitis, oral antibiotics reduced disease severity and shifted the gut microbiota, while ineffective antibiotics did not alter the gut composition, emphasizing the gut’s role in treatment success. Furthermore, research suggests that gut microbiota metabolites, such as short-chain fatty acids (SCFAs) and bile acids, may help to counteract uveitis pathogenesis [40,42]. SCFAs, produced by beneficial gut bacteria, exhibit anti-inflammatory properties and can attenuate ocular inflammation. Decreased levels of secondary bile acids have been observed in uveitis, and their restoration has been shown to ameliorate disease symptoms.

Human studies reveal a shift in the gut microbiome of acute anterior uveitis patients, marked by a rise in harmful, inflammation-promoting bacteria and a corresponding decline in beneficial microbes. This altered microbial profile leads to elevated levels of pro-inflammatory metabolites, contributing to oxidative stress and T-cell dysregulation [43]. Additionally, fecal transplants from individuals with Behçet’s syndrome, a condition associated with uveitis, exacerbated the disease in mice, further implicating gut dysbiosis in uveitis pathogenesis [44].

While further research is needed to fully elucidate the mechanisms involved, these findings highlight the role of gut dysbiosis in the development of uveitis. Understanding the complex interactions between gut microbes and the ocular immune system may pave the way for potential therapeutic approaches, such as microbiome modulation through probiotics, prebiotics, or dietary interventions, offering new hope for the prevention and treatment of this sight-threatening condition.

### 6.2. Gut Dysbiosis and Age-Related Macular Degeneration (AMD)

AMD, the primary cause of irreversible vision loss in developed nations, involves the progressive deterioration of the macula, which is the central part of the retina responsible for sharp, detailed vision [45]. Early in the disease, lipid deposits accumulate beneath the retina, followed by the appearance of drusen, the hallmark yellow deposits that signal the clinical onset of AMD. While AMD is fundamentally a degenerative disease of the retina, it often presents with chronic intraocular inflammation, further complicating its progression. The disease manifests in two forms—“dry AMD”, characterized by the gradual atrophy of retinal cells, and the more aggressive “wet AMD”, involving the abnormal growth of blood vessels that leak fluid and blood, causing severe vision loss [46].

Traditionally, AMD has been attributed to a combination of genetic predisposition, particularly variants in the complement system, and environmental factors such as obesity, smoking, and sun exposure. However, gut dysbiosis has been proposed as a potential culprit in the development of AMD [47]. As the GM is intricately linked to aging and age-related diseases, researchers are actively investigating the role of gut dysbiosis in AMD pathogenesis, although the number of clinical studies remains limited [48].

Animal studies have provided compelling evidence supporting this connection. Mice with induced choroidal neovascularization, a hallmark of wet AMD, exhibit significant shifts in their GM composition compared to healthy counterparts [49]. Moreover, a high glycemic diet has been proven to increase the risk of AMD in mice, while a low glycemic diet offers a protective effect [50]. These observations suggest that dietary factors can modulate the gut microbiome, influencing the development of AMD.

The underlying mechanisms linking gut dysbiosis to AMD are multifaceted. High glycemic conditions can favor the growth of pro-inflammatory bacteria at the expense of beneficial ones, leading to a chronic, low-grade inflammatory state [51]. This inflammation is characterized by elevated levels of pro-inflammatory cytokines and growth factors, thus contributing to the breakdown of the blood–retinal barrier and promoting retinal damage. Conversely, low glycemic conditions can foster a healthier gut microbiome, leading to increased levels of protective metabolites and reduced production of harmful substances, thereby potentially preventing AMD development [52].

In human studies, the picture is less clear, with some discrepancies in findings. While various studies have reported differences in GM composition between AMD patients and healthy controls, the specific microbial taxa and metabolic pathways associated with the disease remain elusive [52,53]. Nevertheless, a reduced *Firmicutes/Bacteroides* ratio appears to be a consistent feature in AMD patients, suggesting a potential microbial signature for the disease [53]. Moreover, alterations in key metabolic pathways, such as glutamate degradation and fatty acid elongation, have been observed in the gut microbiota of AMD patients, further highlighting the potential metabolic link between gut dysbiosis and retinal dysfunction [54].

### 6.3. Gut Dysbiosis and Diabetic Retinopathy

Diabetes mellitus, a prevalent metabolic disorder, often leads to diabetic retinopathy (DR), which is a major cause of blindness [55]. Beyond its systemic effects, diabetes also induces changes in the gut microbiome, contributing to a state of dysbiosis [56]. This imbalance in gut bacteria has been implicated in the development and progression of DR, with obesity-associated dysbiosis exacerbating the condition and interventions like intermittent fasting showing potential protective effects [57].

Studies have revealed specific shifts in the gut microbiome of DR patients, including an increase in *Faecalibacterium*, *Lachnospira*, *Alistipes*, and *Roseburia*, and a decrease in *Blautia*, *Akkermansia*, and *Anaerostipes* [58,59]. These changes are accompanied by alterations in fecal metabolites, with a reduction in key compounds like nicotinic acid and carnosine, vital for mitochondrial function, inflammation regulation, and antioxidant defense [59,60]. Furthermore, a decrease in SCFA-producing bacteria like *Bifidobacterium* and an increase in pro-inflammatory bacteria like *Bacteroidetes* and *Proteobacteria* have been observed in DR patients [61]. These microbial shifts can promote systemic inflammation, potentially contributing to retinal damage.

While the direct causal relationship between gut dysbiosis and DR remains to be fully elucidated, it is crucial to consider the influence of diabetic medications, particularly metformin, on the gut microbiome [62]. Future studies need to account for these confounding factors to accurately assess the contribution of gut dysbiosis to DR pathogenesis.

Additionally, diabetes impacts not only the gut microbiome but also the ocular surface itself, leading to tear film dysfunction, decreased corneal sensitivity, and delayed wound healing [63]. These changes, coupled with potential alterations in the ocular surface microbiota (OSM), may create a vulnerable environment, increasing the risk of ocular infections and further contributing to DR progression [64].

Studies comparing the OSM of diabetic patients with and without DR have revealed differences in microbial composition, with an overrepresentation of *Proteobacteria* and specific genera like *Acinetobacter* and *Escherichia-Shigella* in those with DR [65]. These findings suggest a potential role for both gut and ocular surface dysbiosis in the complex pathogenesis of DR.

### 6.4. Gut Dysbiosis and Dry Eye Disease

Dry eye disease (DED) is a widespread ocular surface disorder affecting millions globally. Its hallmark is a disruption in tear film homeostasis, leading to symptoms such as dryness, discomfort, and even visual disturbances. This imbalance is often caused by excessive tear evaporation, increased tear osmolarity, inflammation, and neurosensory abnormalities. Several factors contribute to DED, including diabetes mellitus, abnormal enzyme metabolism, and decreased mucin secretion—a crucial component for maintaining tear film stability [66].

The meibomian glands, nestled within the eyelids, play a crucial role in ocular surface health by secreting oils that form the outermost layer of the tear film, preventing rapid evaporation. Dysfunction of these glands is a common trigger for evaporative dry eye syndrome. Moreover, the delicate microbial balance on the ocular surface, along with oxidative stress, can contribute to DED. An altered ocular surface microbiota can induce an immune response and generate reactive oxygen species, further destabilizing the tear film and promoting inflammation via inflammasome activation [67].

The GM is crucial in immune system modulation, impacting the balance between pro-inflammatory T-helper 17 (Th17) cells and anti-inflammatory regulatory T cells (Tregs) [68]. Dysbiosis can disrupt this balance, leading to a predominance of Th17 cells and increased production of pro-inflammatory cytokines, such as IL-17. These inflammatory signals can reach the ocular surface through systemic circulation, triggering and perpetuating inflammation in the lacrimal glands and conjunctiva, contributing to DED.

Studies have observed specific shifts in the gut microbiota of individuals with DED, particularly in those with Sjögren’s syndrome, an autoimmune disease often associated with dry eye. These changes include a reduction in beneficial bacteria like *Faecalibacterium* and *Bacteroides* and an overgrowth of potentially harmful bacteria such as *Streptococcus* and *Escherichia/Shigella* [69]. Furthermore, an increase in the abundance of *Prevotella* has been associated with decreased tear secretion and dry eye severity [68].

Gut dysbiosis may also indirectly influence the ocular surface microbiota (OSM), potentially contributing to DED. Studies in mice have shown that antibiotic-induced gut dysbiosis leads to worsened dry eye symptoms and reduced goblet cell density, which are crucial for mucin production and tear film stability [70,71]. Furthermore, an imbalance in the gut microbiota can also affect the levels of short-chain fatty acids, which play a role in reducing inflammation and promoting a healthy ocular surface [71].

### 6.5. Gut Dysbiosis and Glaucoma

Glaucoma, a leading cause of irreversible blindness, is characterized by the progressive degeneration of retinal ganglion cells (RGCs) and their axons, ultimately leading to optic nerve damage [72]. While intraocular pressure (IOP) is a major risk factor, many patients continue to experience vision loss despite IOP control, highlighting the need for therapies targeting alternative mechanisms [73,74,75]. Emerging research suggests a potential role for gut dysbiosis in glaucoma pathogenesis, with a complex interplay between the gut microbiome and ocular health contributing to neurodegeneration.

A crucial link between the gut and glaucoma lies in immune modulation. Studies in mouse models have shown that elevated IOP triggers the infiltration of CD4+ T cells into the retina, leading to neurodegeneration [76]. These T cells are “trained” by the gut microbiota to recognize bacterial heat shock proteins (HSPs) and cross-react with host HSPs, leading to an autoimmune-like response within the eye. Remarkably, germ-free mice lacking gut microbiota did not develop optic nerve damage despite elevated IOP, highlighting the critical role of the gut microbiome in this process [76].

Human studies have also revealed associations between gut dysbiosis and glaucoma. Individuals with chronic irritable bowel syndrome, a condition often linked to gut dysbiosis, exhibit a higher risk of developing glaucoma, further supporting the gut–eye axis hypothesis [77]. Analysis of fecal microbiota in glaucoma patients has shown an enrichment in pro-inflammatory bacteria like *Enterobacteriaceae*, *Prevotellaceae*, and *Escherichia coli*, and a depletion in beneficial bacteria such as *Megamonas*, *Blautia*, and *Fusicatenibacter* [78]. These alterations suggest a potential contribution of gut dysbiosis to the neuroinflammatory processes involved in glaucoma.

Beyond immune modulation, gut dysbiosis may influence glaucoma through metabolic changes. Some studies report alterations in gut-derived metabolites, such as increased isocitrate and citric acid or reduced citric acid, in glaucoma patients [78]. While the exact implications remain unclear, these findings suggest a potential metabolic link between the gut and the eye.

Additionally, butyrate, a beneficial SCFA produced by gut bacteria, has been shown to lower IOP in rats, suggesting a potential protective role [79]. Conversely, an increase in trimethylamine, a uremic toxin produced by the gut microbiome, has been observed in the aqueous humor of glaucoma patients, suggesting a potential detrimental effect [80].

Furthermore, changes in the ocular surface microbiota (OSM) have also been reported in glaucoma patients. Studies have observed increased abundance of certain bacteria, including *Streptococcus*, *Staphylococcus*, and *Corynebacterium* on the ocular surface of individuals with glaucoma [81]. These alterations in the OSM may contribute to ocular surface inflammation and potentially impact disease progression.

## 7. Therapeutic Modulation of the Gut Microbiome

The understanding of the gut microbiome’s role in various systemic and ocular diseases has sparked interest in therapeutic approaches to restore a healthy microbial balance or eubiosis.

### 7.1. Probiotics: Harnessing Beneficial Bacteria

Probiotics, defined as live microorganisms that provide health benefits when consumed in appropriate quantities, present a promising avenue for combating gut dysbiosis and the ocular complications it can trigger. The most frequently employed probiotics are members of the *Lactobacillus* and *Bifidobacterium* genera, which are often found to be diminished in a range of gastrointestinal and systemic diseases [82,83,84]. These beneficial microbes exert their positive influence through a variety of mechanisms.

Notably, probiotics play a crucial role in curbing inflammation. They achieve this by modulating key signaling pathways within the gut epithelial cells, such as JAK/STAT and NF-kB, thereby promoting tissue healing and bolstering the cellular response to stress [85]. Additionally, they extend their regulatory reach to the immune system, influencing the activity of diverse immune cells including T cells, dendritic cells, and macrophages, thus ensuring a balanced immune response [86,87].

Probiotics also contribute to the fortification of the gut barrier, a critical line of defense against the leakage of harmful substances and bacteria into the bloodstream. They stimulate the production of mucus, a protective layer lining the gut, and strengthen the tight junctions between epithelial cells, enhancing the barrier’s integrity [88,89]. Moreover, these beneficial microbes possess the ability to produce bacteriocins and other antimicrobial peptides, natural weapons that directly target and inhibit the growth of pathogens, further bolstering gut health and immune function [90].

These beneficial effects of probiotics have been observed in various disease models, including ocular conditions. In mice with ocular inflammation, applying eye drops containing the cell-free extract of the probiotic *Lactiplantibacillus plantarum* led to reduction in pro-inflammatory cytokine levels [91]. In another study, administration of *Escherichia coli O83:K24:H31* to mice with autoimmune uveitis led to decreased T-cell immunoreactivity and increased production of antimicrobial peptides, effectively blocking disease progression [92].

Human studies, although limited, also show promising results. A case report demonstrated the potential benefit of combining steroids with a probiotic mixture in a patient with refractory acute anterior uveitis [93]. Additionally, pilot studies in adults and children with chalazion showed that adding a probiotic blend to the standard treatment shortened healing time [94,95].

In the context of dry eye disease, probiotics like *IRT5* (a mixture of *Lactobacillus* and *Bifidobacterium* strains) have shown efficacy in restoring tear secretion and modulating immune responses in mouse models [96]. Oral administration of *Bifidobacterium bifidum* and *Lactiplantibacillus plantarum* also improved tear production, goblet cell density, and ocular inflammatory parameters in mice with dry eye [97].

Furthermore, engineered probiotics expressing beneficial molecules like ACE2 and Ang-(1-7) have demonstrated promising results in mitigating diabetic retinopathy in mice [98,99]. These engineered probiotics reduced inflammation, improved blood–retinal barrier function, and protected retinal ganglion cells.

### 7.2. Prebiotics and Synbiotics: Nourishing the Gut’s Beneficial Microbes

Prebiotics, a category of non-digestible carbohydrates abundant in fiber, serve as nourishment for beneficial gut microbes, fostering a healthier microbial community and offering potential therapeutic advantages for the host [100]. Examples of prebiotics include galacto-oligosaccharides, fructans, inulin, and various other glucose-derived oligosaccharides and starches [101].

These substances selectively promote the growth of anti-inflammatory bacteria within the gut, contributing to a balanced microbial ecosystem. This targeted promotion has led to the exploration of prebiotics as preventive or therapeutic agents for various health problems, such as neurological disorders, cardiovascular and skin diseases, and Chron’s disease [102,103,104,105].

While research into prebiotics’ impact on eye health is still in its early stages, there are promising developments. Innovative contact lens formulations incorporating the prebiotic resveratrol have demonstrated a potential growth reduction of harmful biofilm-forming bacteria and decreased inflammation on the ocular surface [106]. Additionally, studies suggest that the beneficial effects of hyaluronan, a common component of eye drops, may be partly attributed to its prebiotic activity, fostering a healthier ocular surface microbiota [107].

A diet rich in prebiotics, particularly those that promote butyrate-producing bacteria, may alleviate dry eye symptoms in patients with Sjögren’s syndrome [108]. Although the exact mechanisms require further exploration, these findings suggest a potential link between gut microbiome modulation and improved ocular health.

While the direct benefits of prebiotics alone in eye disorders remain to be fully elucidated, combining them with probiotics (synbiotics) has shown promising results in clinical studies. In a case study, a synbiotic formulation reduced clinical symptoms and inflammatory markers in a uveitis patient [109]. Similarly, a randomized controlled trial demonstrated that a synbiotic mixture improved tear secretion and ocular surface health in patients suffering from dry eye disease [110].

Overall, while research is still limited, the use of prebiotics and synbiotics in managing ocular conditions linked to gut dysbiosis presents an exciting avenue for future investigation. Unraveling the complex relationship between prebiotics, the gut microbiome, and ocular health may lead to innovative therapeutic strategies for a variety of eye diseases.

### 7.3. Faecal Microbiota Transplantation (FMT)

Fecal microbiota transplantation is the process of transferring gut microbes from a healthy donor to a recipient and it offers a radical yet promising approach to restoring gut microbial balance [111]. Traditionally administered via colonoscopy, less invasive methods like enemas, nasogastric tubes, or oral capsules are gaining traction [112,113].

Beyond its established success in treating recurrent *Clostridium difficile* infections, FMT is now being explored for a range of conditions, from Crohn’s disease and irritable bowel syndrome to obesity and neurodegenerative disorders [107,108,111,114,115,116,117,118,119,120,121,122]. The benefits of FMT include re-establishing a healthy gut microbiota, reducing inflammation, increasing the production of beneficial short-chain fatty acids, and improving gut barrier integrity [123,124].

The potential of FMT in modulating the gut–eye axis and addressing ocular diseases is an area of growing interest. Studies in mice have shown that FMT can restore ocular surface immunity and reduce dry eye symptoms in models of Sjögren’s syndrome [125,126]. Germ-free and antibiotic-treated mice, which exhibit impaired ocular surface immunity and increased susceptibility to infection, showed improvements in corneal barrier function and reduced inflammation following FMTs from healthy donors.

Furthermore, FMT has been proven to influence retinal health. Transferring gut microbiota from old mice to young mice increased intestinal permeability and retinal inflammation, while the reverse transfer had a protective effect [127]. This suggests that FMT may modulate the gut–eye axis and impact age-related ocular changes.

While human studies are limited, a small clinical trial demonstrated that FMT could improve dry eye symptoms in patients with Sjögren’s syndrome [128]. Alterations in gut fungal diversity have been observed in uveitis patients, highlighting the need to consider the entire gut microbiome for maximizing FMT efficacy [129].

## 8. Conclusions

The gut microbiome, a complex and dynamic ecosystem, exerts a profound influence on human health, extending far beyond its traditional role in digestion. Through intricate mechanisms involving metabolism, immune modulation, gut barrier function, and the gut–brain axis, the gut microbiota contributes to overall well-being. Disruptions to this delicate balance, known as dysbiosis, can trigger a cascade of health complications, including various ocular diseases.

Emerging research has highlighted the gut–eye axis, a communication pathway linking the gut microbiome to ocular health. Dysbiosis can impact ocular immunity, inflammation, and the blood–retinal barrier, contributing to conditions like uveitis, AMD, diabetic retinopathy, dry eye disease, and glaucoma. While the precise mechanisms remain an active area of investigation, therapeutic modulation of the gut microbiome through probiotics, prebiotics, synbiotics, and FMT offers promising avenues for managing and preventing these ocular diseases. Future research focused on unraveling the intricacies of the gut–eye axis holds the potential to revolutionize our understanding and treatment of ocular diseases, ultimately improving vision and quality of life for countless individuals.

## Data Availability

Data is available upon request from the corresponding author.
